# The Uniqueness of Clear Cell Renal Cell Carcinoma: Summary of the Process and Abnormality of Glucose Metabolism and Lipid Metabolism in ccRCC

**DOI:** 10.3389/fonc.2021.727778

**Published:** 2021-09-15

**Authors:** Xiaochen Qi, Quanlin Li, Xiangyu Che, Qifei Wang, Guangzhen Wu

**Affiliations:** Department of Urology, The First Affiliated Hospital of Dalian Medical University, Dalian, China

**Keywords:** lipid metabolism, clear cell renal cell carcinoma, Warburg effect, glucose metabolism, cholesterol

## Abstract

Kidney cancer is a cancer with an increasing incidence in recent years. Clear cell renal cell carcinoma (ccRCC) accounts for up to 80% of all kidney cancers. The understanding of the pathogenesis, tumor progression, and metastasis of renal carcinoma is not yet perfect. Kidney cancer has some characteristics that distinguish it from other cancers, and the metabolic aspect is the most obvious. The specificity of glucose and lipid metabolism in kidney cancer cells has also led to its being studied as a metabolic disease. As the most common type of kidney cancer, ccRCC has many characteristics that represent the specificity of kidney cancer. There are features that we are very concerned about, including the presence of lipid droplets in cells and the obesity paradox. These two points are closely related to glucose metabolism and lipid metabolism. Therefore, we hope to explore whether metabolic changes affect the occurrence and development of kidney cancer by looking for evidence of changes on expression at the genomic and protein levels in glucose metabolism and lipid metabolism in ccRCC. We begin with the representative phenomenon of abnormal cancer metabolism: the Warburg effect, through the collection of popular metabolic pathways and related genes in the last decade, as well as some research hotspots, including the role of ferroptosis and glutamine in cancer, systematically elaborated the factors affecting the incidence and metastasis of kidney cancer. This review also identifies the similarities and differences between kidney cancer and other cancers in order to lay a theoretical foundation and provide a valid hypothesis for future research.

## Introduction

According to the Global Cancer Statistics for 2020, renal cancer is a common cancer that accounts for 2.2% of the total cancer incidence and 1.8% of the total cancer mortality (1). In recent years, the incidence of renal cancer has been increasing annually, which is closely related to the improvement in the quality of life of people in modern society. Common carcinogenic factors such as cigarette smoking, hypertension, obesity, occupation, and radiation are the main causes of renal cancer. Clear cell renal cell carcinoma (ccRCC) is the most common type of renal cell carcinoma, accounting for 75%–85% of all renal cell carcinoma patients (2).

Currently, renal cell carcinoma (RCC), including ccRCC, is generally considered a metabolic disease. An obvious feature of renal cell carcinoma is the target gene mutation involved in metabolic pathways. These include mutations in the regulatory genes involved in aerobic glycolysis, fatty acid metabolism, and tryptophan glutamine utilization (3). With the discovery of the Warburg effect 80 years ago, studies on the abnormal metabolism of cancer cells began to increase. The Warburg effect indicates that abnormal aerobic glycolysis occurs in cancer cells, producing lactic acid and pyruvate. The Warburg effect has been proven to be significantly more obvious in ccRCC than in ordinary tissues (4). The end product of pyruvate in the body is acetyl-CoA, which is the raw material for cholesterol, a type of lipid (5). Although this energy rearrangement is not present in all cancers, renal cell carcinoma is thought to be driven by metabolic changes due to the high rate of mutations in genes that control metabolism, such as von Hippel-Lindau (VHL) in the hypoxia pathway, mammalian target of rapamycin (mTOR), phosphatase and tensin homolog deleted on chromosome ten (cf), and mesenchymal epithelial transition factor in the PI3K (phosphatidylinositol-3-kinase) -Akt (protein kinase B) -mTOR pathway (6). Therefore, abnormal lipid metabolism is known to occur in renal cancer cells. A large amount of lipid accumulation in ccRCC cells provide the evidence of the abnormal lipid metabolism. Studies have shown that the contents of cholesterol, cholesterol esters, and neutral lipids (triglycerides) in ccRCC cells are much higher than those in ordinary tissues (7).In addition to differences in lipid metabolism, there are other notable differences between ccRCC and other tumors, such as the obesity paradox (8), in which obese kidney cancer patients have a higher incidence, but with a better prognosis of kidney cancer than lean people (9). Furthermore,

Recent research has shown that there is a correlation between abnormal lipid metabolism and ferroptosis. This may be due to the fact that a mechanism of ferroptosis is under the action of iron divalent or ester oxygenase, catalyzing the highly expressed unsaturated fatty acids on the cell membrane, facilitating lipid peroxidation, and inducing cell death (10). Concurrently, glutamine metabolism has long been considered to be related to the development of cancer. As a substitute for cancer cells, glutamine provides carbon sources for the tricarboxylic acid cycle (TCA) cycle and provides a large amount of raw materials for the synthesis of abnormal lipids, we believe that it is associated with ccRCC (11). Glutathione, the metabolite of glutamine, is an important participant in the body’s antioxidant system (12). Is the abnormal metabolism of glutamine related to the occurrence and development of cancer cells?

All these abnormal phenomena point to the problem of lipid metabolism in ccRCC. Therefore, this review emphasizes the relationship and significance of lipid metabolism in ccRCC in the occurrence, development, and treatment of cancer.

## Warburg Effect: Provide a Large Amount of Acetyl-CoA for Lipid Metabolism

**Figure 1:**
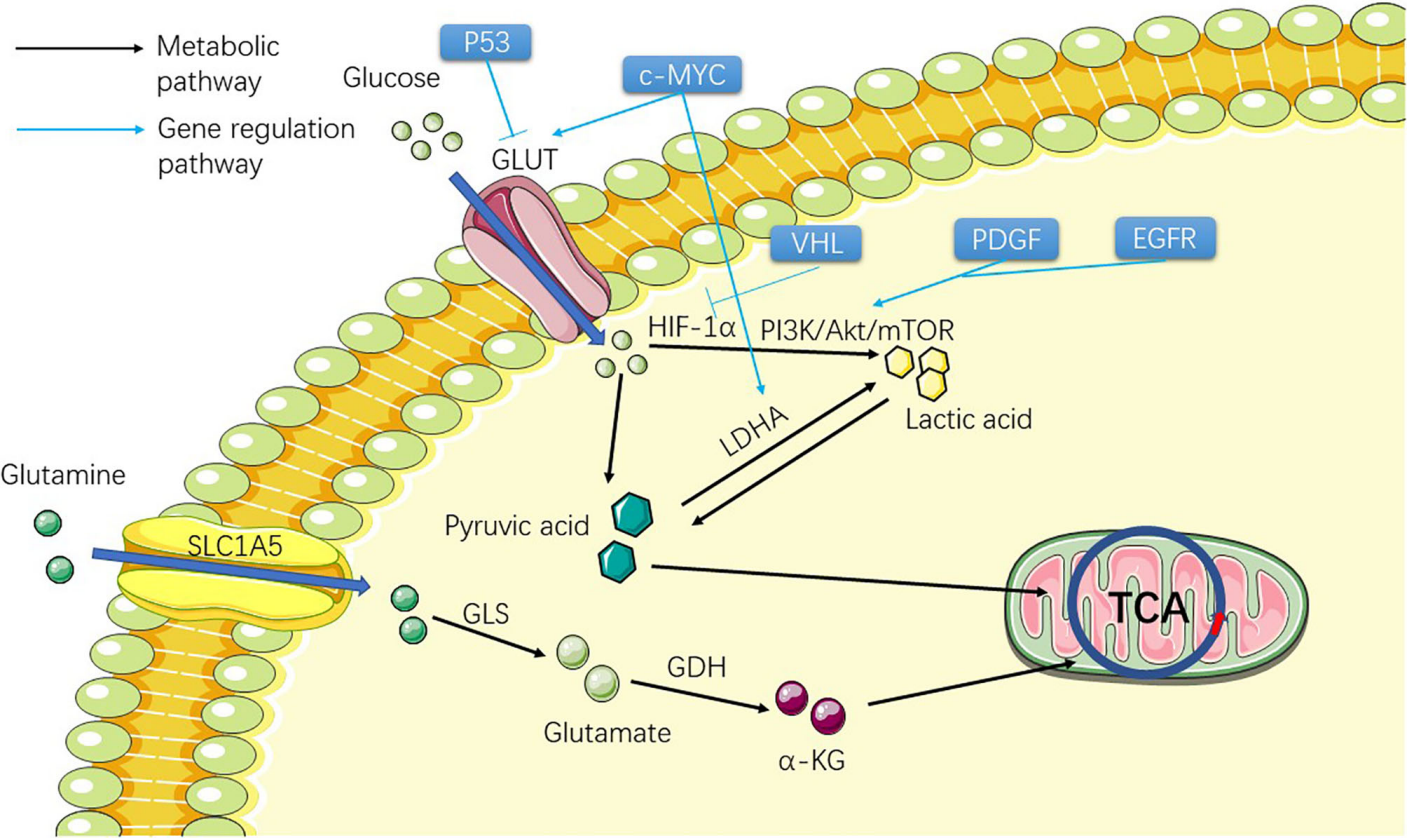
Figure shows the metabolic process of the substances involved in the Warburg effect in ccRCC cells before TCA, including two substances, glucose and glutamine. The picture shows the process of the two substances entering the cell from the outside of the cell and transforming them into substances that can participate in TCA through metabolism inside the cell, providing raw materials for the subsequent lipid metabolism.

Mutations in genes related to aerobic glycolysis and metabolism are major risk factors for renal cell carcinoma. For example, *VHL* and protein 53 (P53) have been found to have mutations in RCC and their role in promoting the progression of RCC is extremely obvious (13). The decreased expression of VHL in RCC can upregulate hypoxia-induced vascular endothelial growth factor (VEGF), and VEGF can guarantee the nutritional supply of tumor cells by promoting angiogenesis in tumors (14). *The tumor protein 53 (TP53)* mutation also resulted in fewer renal tumor cells dying from oxidative damage (15). Both genes regulate oxidative processes in the body and directly or indirectly affect the oxidation of glucose. Metabolic rearrangement takes place in cancer cells, including the production of lactic acid by cancer cells through aerobic glycolysis, which replaces the tricarboxylic acid cycle that uses glucose in normal cells (16). This change makes cancer cells use glucose much more efficiently than normal cells, a condition known as the Warburg effect and a well-established feature of cancer energy metabolism (17) (**Figure 1**).

Current studies have shown that this is particularly evident in ccRCC (4). Previous studies have shown a link between elevated lactic acid levels and the progression of cancer metastasis (18–20). Lactic acid also stimulates endothelial cells to produce VEGF (21). Meanwhile, lactic acid has been shown to promote cancer cell metastasis through the transforming growth factor-β2 (TGF-β2) pathway (22). Aerobic glycolysis inefficiently produces adenosine triphosphate (ATP), but confers many advantages to tumor cells. One of the products, lactic acid, can also be converted to pyruvate to provide a raw material for the synthesis of acetyl-CoA (23). This effect provides a large amount of fatty acids and cholesterol for cancer cells and a large number of raw materials that aid in cancer cells proliferation and metastasis. This process of cancer cells is regulated by many oncogenes or oncogenic pathways, such as MYC (myelocytomatosis oncogene), P53, hypoxia inducible factor-1α, 2α (HIF-1α, HIF-2α), AMPK (adenosine 5’-monophosphate-activated protein kinase)/mTOR signal pathway, and PI3K/Akt signaling pathway (24).

**Figure 2:**
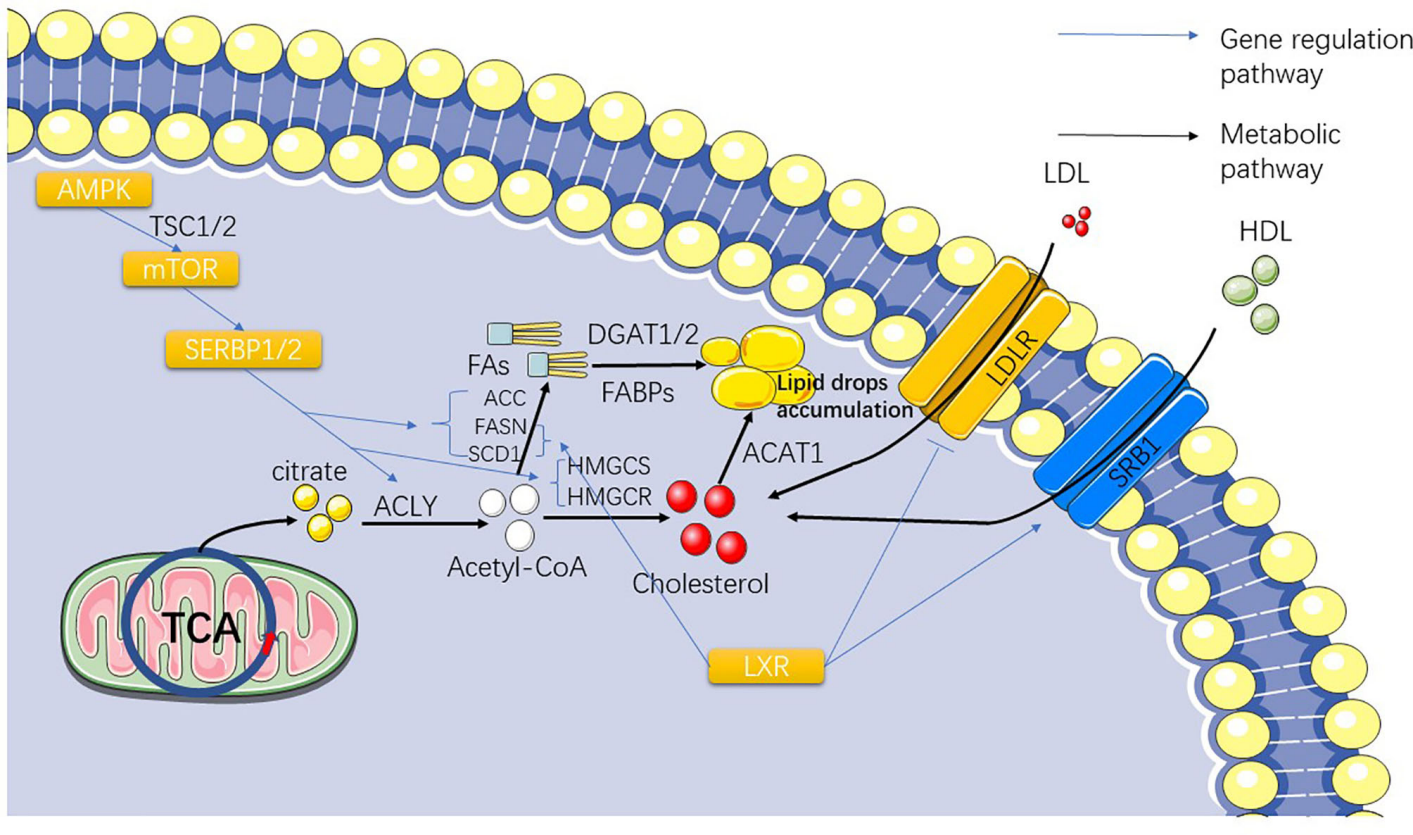
The figure shows the gradual conversion of citrate to lipid and cholesterol after TCA. The influence of the SERBP family and LXR on the synthesis and exocytosis of lipid acids and cholesterol are also presented.

Glucose transporter (GLUT) mutation in ccRCC allows ccRCC cells to obtain large amounts of glucose from extracellular to support cell growth and metastasis (25). Therefore, many genes regulating GLUT family mutation are theoretically associated with the development of ccRCC. c-MYC (**Table 1**) (26) can directly transcriptionally activate glycolytic genes encoding glucose transporter 1 (GLUT1) (27) and lactate dehydrogenase A (LDHA) and promote abnormal glycolysis. Studies have suggested that ccRCC is related to the up-regulation of *c-MYC *(28). P53 (**Table 1**) (29) tumor suppressor directly inhibits the transcription of GLUT1 and GLUT4 genes, thereby inhibiting the uptake of grape and lactic acid production (**Figure 1**). The function of P53 also leads to its down-regulation related to ccRCC (30). HIF-1α (31) promotes glycolytic gene expression by binding to hypoxia response elements on glycolytic gene promoters. Under hypoxia, the degradation of the HIF-1α subunit is inhibited, and the active HIF-1 is transferred to the nucleus to regulate the transcription of many genes (**Figure 1**). These include many of the genes involved in tumorigenesis: VEGF, platelet-derived growth factor (PDGF), aldolase A, enolase 1, and LDHA, which regulate tumor angiogenesis and the process of abnormal glycolysis (32, 33). HIF-2α is involved in angiogenesis and several other processes and is thought to activate cancer progression (34). At present, some experiments have been conducted to inhibit ccRCC progression by inhibiting the angiogenesis of HIF-2α, and some results have been obtained (35) (**Figure 1**). In this study, the HIF-2 antagonist PT2399 was used to observe the expression of HIF-2 downstream proto-oncogenes (VEGF, LDHA, etc.) in tumor cells. The results showed that the expression of these downstream proto-oncogenes was significantly reduced. *VHL* (**Table 1**), which regulates *HIF-1α* and *2α* and other types of cancer-related genes, has been shown to be extensively absent in renal cancer cells (36) (**Figure 1**). A review showed that 91% of patients with ccRCC had a deletion of *VHL* by methylation or mutation (6). Harlander et al. proved that VHL combined with *TP53* and *retinoblastoma susceptibility gene 1* mutation could lead to the occurrence of ccRCC in mice (13). The accumulation of *HIF-α* was caused by a loss of *VHL*. Nevertheless, *HIF* activation also affects ROS in two directions: 1. It affects the TCA in mitochondria to reduce ROS production; 2. Activation of downstream target gene 3 *NADPH (nicotinamide adenine dinucleotide phosphate) oxidase (NOX)* to increase ROS production, and increased ROS levels may also be a contributing factor to HIF stabilization during hypoxia and reoxygenation (37). A large number of HIF-α enters the nucleus and activates the transcription of HIF target genes, which affects several oncogenic pathways, including VEGF, GLUT1 (glucose transporter type I), and erythropoietin (38, 39). Hoefflin et al. demonstrated the oncogenic role of *HIF-1α* and *HIF-2α* in the initiation of ccRCC using a mouse ccRCC model and suggested that changes in the balance of *HIF-1α* and *HIF-2α* activity may influence different aspects of ccRCC biology and disease aggressiveness (40). *Epidermal growth factor receptor (EGFR)* (**Table 1**) (41), *PDGF *(39), and some of their downstream pathways have been shown to regulate the Warburg effect by affecting the PI3K/Akt/mTOR pathway (**Figure 1**). The AMPK (**Table 1**)/mTOR pathway is an important pathway for the regulation of cell metabolism and plays an important role in cancer cells (42, 43). *Tuberous sclerosis complex subunit 1/2 (TSC1/TSC2)* (**Figure 2**) is an important gene in this pathway, and the latter has been proven to be correlated with ccRCC through studies on the relationship between the tuberous sclerosis complex and ccRCC (44, 45). The products of aerobic glycolysis provide energy to cancer cells, while generating a large number of by-products and lactic acid (46). Glucose is broken down by glycolysis into pyruvate, which is eventually converted to acetyl-CoA (47). So far, everything seems to be consistent with what happens in normal cancer cells when a gene mutation occurs: the mutation genes that regulates GLUT causes a lot of glucose to enter the ccRCC cells. In the case of down-regulation or even disappearance of VHL expression, influenced by HIF and PI3K/Akt/mTOR pathway, glucose abnormal metabolism forms lactic acid and provides energy for cells. The outcome of lactic acid is generally to leave the cells. How does this provide raw material for the subsequent lipid metabolism.

**Table 1:** Upstream gene of lipid metabolism.

gene	The influence of metabolic pathways	Relevance of RCC
c-MYC	Upregulation of the expression of GLUT1 and LDHA	Upregulation is a risk factor for RCC
	Upregulation of glucose intake and lactic acid production	
P53	Downregulation of the GLUT family (GLUT1/4) results in a decrease in glucose intake	Downregulation is a risk factor for RCC
VHL	Inhibition of HIF-1α and HIF-2α activation, Down-regulation of gene expression	Inhibition or deletion of expression is often found in RCC
	Upregulation of anaerobic glycolysis and promotion angiogenesis in cancer tissues	
EGFR	Promotion of the Warburg effect in cancer cells through the PI3K/Akt/mTOR pathway	Upregulation is a risk factor for RCC
AMPK	Regulation of mTOR pathway affects glycolysis	Significant reduction in expression was observed in RCC
	Upregulation of SERBP affects the synthesis of fatty acids and cholesterol.	
SREBP	Various synthases and rate-limiting enzymes that regulate the synthesis of acetyl-CoA and the conversion pathways to cholesterol and fatty acids	Complex relationship with RCC
LXR	Regulation of cholesterol intake receptor LDLR and SRB1, as well as genes regulating fatty acid synthesis such as SCD-1 and FASN	Both positive and negative regulation of cholesterol intake and fatty acid synthesis in RCC cells were observed

There is a reciprocal transformation between lactic acid and pyruvic acid in ccRCC cells. LDHA catalyzes the conversion of pyruvate to lactic acid, which is a reversible reaction (**Figure 1**). The phosphorylation of LDHA is closely related to cancer development. Anoikis is a specific form of programmed cell death induced by the loss of contact between cells, the extracellular matrix, and other cells. It plays an important role in the homeostasis of developmental tissues in balancing disease development and tumor metastasis (48). A recent study sheds light on the role of LDHA in the development of anoikis, which leads to apoptosis in cancer cells (49). In this study, we used shRNA to inhibit LDHA activity to make cancer cells sensitive to anoikis induction. This leads to a decrease in cell invasiveness. One type of catalytic subtype of the LDHA mutant, Y10F, expresses phosphorus-deficient LDHA and has the same effect (49). Concurrently, LDHA knockout can also lead to increased mitochondrial respiration, which reduces the ability of cells to proliferate under hypoxic conditions and inhibits the occurrence of tumors (50). Previously, the influence of mitochondrial DNA copy number alteration on the occurrence and development of RCC has shown that there is higher expression of LDHA in RCC tissues than in the corresponding normal renal tissues (51), suggesting that LDHA promotes the occurrence and progression of RCC as an oncogene. Meanwhile, studies have applied LDHA inhibitors to the treatment of renal cell carcinoma and summarized the basic mechanism of treatment by observing the clinical indicators of the object. The abnormal expression of LDHA after siRNA treatment changed the expression of the cell cycle and apoptosis-related proteins and significantly reduced the migration and invasion ability of cancer cells (52). However, the results cannot directly explain the effect of LDHA on RCC. In terms of the Warburg effect of immune cells, a study on the relationship between T effector cells and Warburg in January of the same year pointed out that there is a positive feedback loop between the LDHA and the PI3K/Akt signaling pathway in the process of T effector cell maturation, which makes glycolytic ATP act as a rheostat to measure and modulate PI3K-dependent signals (53). LDHA has been shown to act as a downstream signal of oncogenes to regulate glycolysis and carcinogenesis in ccRCC (54). Therefore, its role in activating the Warburg effect in T effector cells is supported by some evidence. In view of the importance of LDHA in the Warburg effect, we believe that the orientation of LDHA in the catalytic conversion of pyruvate and lactic acid in renal cancer is the focus of current research. Both lactic acid and pyruvate are valuable for ccRCC. It includes the pathological characteristics of ccRCC cells and metastasis of ccRCC. Therefore, it is important to identify the advantages and disadvantages of the mutual transformation of the two, including early targeted therapy and the prevention of metastasis.

Notably, a new study in April 2021 offered a radically different view of cancer cell metabolism: cancer cells do not overconsume glucose (55). Glucose was found to be mainly supplied to the immune-infiltrating cells surrounding the cancer cells, while glutamine and lipids were largely delivered to the cancer cells. Fortunately, the study used ccRCC as subjects, and the results showed that the glucose supply in ccRCC was given priority to immune cells rather than cancer cells. This experiment revealed a contradictory view of the Warburg effect: there is no one type of cell that is receiving too much or too little of a nutrient; they are all programmed to take in different nutrients. The ideas are too new to be supported by other studies, but this does not mean that it cannot overturn what has been established about how cancer cells metabolize. We hope that similar studies will follow.

## Abnormal Metabolism of Fatty Acids and Cholesterol

We know from the Warburg effect that an increased amount of acetyl-CoA is produced in cancer cells through aerobic glycolysis and how acetyl-CoA, a compound for cholesterol and lipid synthesis, turns into fatty acids and cholesterol.

With increasing evidence that the phenotype of ccRCC (and to a lesser extent its genetic profile) is similar to that of adipocytes, the theoretical basis for targeting lipid synthesis pathways has taken root (56–58). The metabolic abnormalities of the whole cancer cell go from acetyl-CoA to abnormal lipid metabolism. Fatty acid synthesis depends on acetyl-CoA, and normal cells generated through the Krebs cycle can meet the demand of basic cellular functions, but mutations of stearoyl-CoA desaturase 1 (SCD1), fatty acid synthase (FASN), and acetyl-CoA carboxylase (ACC) in ccRCC can lead to the emergence of a large number of abnormal pathways for the synthesis of acetyl-CoA and synthetic fatty acid (FA), which are ultimately stored in the form of lipid droplets in the cytoplasm (24, 59, 60) (**Figure 2**). This also explains the existence of lipid droplets in ccRCC cells. Therefore, in order to understand abnormal lipid metabolism in ccRCC, it is necessary to identify the genes that control abnormal fatty acid synthesis.

SCD1 is an important regulator of lipid synthesis, mainly regulated by serpine mRNA binding protein-1 (SERBP-1) (**Table 1**) (61) (**Figure 2**). *SCD1* regulates the transformation of saturated fatty acids into monounsaturated fatty acids in cells and is believed to be closely related to lipid metabolism in cancer (62, 63). Currently, it has been reported that SCD1, a downstream gene of the AMPK-mTOR-SERBP1 signaling pathway, is the target gene of sorafenib (64). Similar to the role of *SCD1*, *FASN* and *ACC* both play important regulatory roles in lipid synthesis in ccRCC cells (**Figure 2**). These three genes exhibit higher mRNA expression in ccRCC cells than in normal cells, and FASN has been associated with poor prognosis in patients (65, 66). FASN dominates the synthesis of lipids in cells, and it has been considered as an important target of anticancer targeted drugs as early as 2007 (61); however, subsequent drug-related experiments and clinical applications are not satisfactory (67, 68). Studies have shown that FASN is upregulated in ccRCC, which is significantly correlated with the presence of lipid droplets in ccRCC cells (69). The study also showed that FASN is an independent predictor of poor survival in ccRCC patients, which also indicates that the upregulation of FASN plays a promoting role in the progression of ccRCC (69). Acetyl-CoA carboxylase catalyzes ATP-dependent acetyl-CoA carboxylation, a rate-limiting step in fatty acid biosynthetic (70). In 2017, studies reported the use of TGFβ-activated kinase to phosphorylate ACC to prevent the metastasis and recurrence of breast cancer (71). In 2019, a study showed that inhibiting ACC expression in HCC cells by phosphorylation and the use of ND-654, an inhibitor of ACC, can slow down cancer progression (72). Theoretically, the carboxylation of acetyl-CoA related to lipid synthesis plays a decisive role in the occurrence and development of ccRCC. We also hypothesized that ACC is correlated with ccRCC. A 2013 article reported that ACC upregulation leads to worse outcomes in patients with RCC (73). Currently, there have been reports of *in vitro* inhibition of RCC cell lines (786-O) by TOFA, an inhibitor of ACC, which was speculated to be related to the inhibition of the PI3K/Akt/mTOR pathway (68). Currently, treatments for ACC have not been widely used in the treatment of RCC, but we believe that ACC-related treatments are still a useful tool for the treatment of RCC.

In addition to regulating the synthesis of fatty acids by acetyl-CoA, as the precursor of acetyl-CoA synthesis, citric acid depends on a very important enzyme, ATP citrate lyase (ACLY), which synthesizes the target product (74) (**Figure 2**). *ACLY* is a downstream target of *SREBPs (* 75) and has been found to be upregulated in a variety of cancers, including breast cancer, non-small cell lung cancer, and hepatocellular carcinoma (76, 77). Nevertheless, a bioinformatics analysis of ccRCC-associated gene pathways suggests that *ACLY* is associated with favorable outcomes in ccRCC patients [*p*<0.001 (73)]. However, after finding more research reports related to the expression level of ACLY in ccRCC, we found that most of the studies indicated that ACLY was highly expressed in ccRCC (66, 78). This result confirms that the role of *ACLY* in patients with ccRCC is not simple. Early inhibitors targeting *ACLY* have been less active and have generally been less widely used in the clinic, but a new 2019 study has broadened the approach to ACLY inhibitors (79). They observed that an allosteric site at the citric acid binding site of the ACLY inhibitor NDI-091143 increased the efficacy of the ACLY inhibitor. Although the study of ACLY in ccRCC is limited to the comparison of its gene expression levels in different samples to prove the high expression of ACLY in ccRCC samples, several clues have proved that the treatment of ACLY can be applied to the clinical treatment of ccRCC. However, from the functional analysis of ACLY, acetyl-CoA can also be converted into oxaloacetic acid (80), which indirectly leads to the inhibition of the reaction of fatty acid synthesis and may be the cause of this contradiction. Acetyl-CoA synthetase 2 (ACSS2), a conservative ribozyme that converts acetic acid into acetyl-CoA, has attracted our attention (81). *ACSS2* is associated with renal cancer metastasis and poor prognosis, which has been confirmed at the genetic level (82, 83) (**Figure 2**). Studies have shown that the upregulation of *ACSS2* expression enhances the migration and invasion of RCC cells, resulting in a poor prognosis. This is mainly achieved through the PI3K/Akt pathway. Nonetheless, a study on the relationship between *ACSS2* and cancer has shown that *ACSS2*-mediated histone acetylation plays an important role in maintaining cell homeostasis and tumor development, providing a clearer path for the research of *ACSS2* (84). It is also a new idea to explore the therapy of ccRCC and other cancers that target this gene (85, 86). Based on the above study on the application value of ACLY and *ACSS2* in cancer treatment, we believe that it is highly practical to develop new treatment methods based on these two genes.

Hydroxymethylglutaryl-CoA synthase (HMGCS) and hydroxymethylglutaryl-CoA reductase (HMGCR) are synthases of the cholesterol synthesis intermediate HMG-CoA, in which HMGCR is the target gene for statins (**Figure 2**). Statins are mainly used in the clinical prevention of coronary heart disease and other diseases related to lipid accumulation (87). A study showed that statins have a significant therapeutic effect on VHL-deficient ccRCC (88), but a population-based case-control study showed no significant effect of long-term use of statins on the prevention of renal cancer (89). If we pay attention to the mechanisms of the two genes and the major role of cholesterol in kidney cancer independently, we can immediately assume that they are present as a risk gene in kidney cancer cells, which is indeed the case in many other cancers (90). Interestingly, after collecting and analyzing the expression data of *HMGCR* and *HMGCS* from the TCGA database in the Pan-Cancer Project, we found that they actually exist in the form of protective genes in kidney cancer, which is completely different from other cancers (91). Our previous research on this situation sheds some light.

After understanding the synthesis of cholesterol and fatty acids in ccRCC, another important question arises: Do cholesterol and fatty acids in ccRCC depend solely on the intracellular synthesis? Is there a need for extracellular uptake? Liver X receptor (**Table 1**) (LXR) is a nuclear transcription factor receptor that regulates a number of key molecules involved in fatty acid synthesis and cholesterol transport. LXR regulates many downstream genes related to cholesterol and lipid metabolism, including *SCD-1*, *FASN*, *SREBP*, *LDLR*, and adenosine triphosphate binding cassette transporter A1 (ABCA1) and so on (92, 93) (**Figure 2**). By studying the agonists of LXR and the reverse agonists SR9243 and LXR623, we found that both of them can kill ccRCC cells (93). However, their mechanisms differ. SR9243, as an LXR inhibitor, downregulates *SREBP-1c*, *ACC*, *FASN*, and *SCD1*, while other FA synthesis genes inhibit the synthesis of FA in CCRCC cells. LXR623 as an inverse agonist that upregulates the cholesterol exogenous genes *ABCA1* and *MYLIP*, which encodes one type of E3 ubiquitin ligase, resulting in the inability of cholesterol accumulation in cancer cells. We also found an interesting phenomenon in the experiment that the expression of genes regulating fatty acid synthesis such as *FASN*, *SCD-1*, and *SREBP* in ccRCC was relatively increased, but the expression of important genes regulating cholesterol synthesis, such as HMGCR and HMGCS, was downregulated. We hypothesized that this might represent the high presence of cholesterol in ccRCC cells due to exogenous uptake rather than autosynthesis. Later, we found that LXR could upregulate *SRB1* (responsible for HDL absorption) and downregulate *LDLR* (responsible for LDL absorption). It was also speculated that ccRCC guaranteed intracellular cholesterol content through a large intake of high- density lipoprotein. A recent study on the risk reduction of ccRCC by *LDLR* variants also demonstrated the downregulation of *LDLR* gene expression in ccRCC cells (94). These experimental results also showed that the production and accumulation of fatty acids and cholesterol in ccRCC were variable, and we could not treat their beneficial or harmful side in isolation. We found that in renal cell carcinoma, the diversity and uncertainty of many related regulatory genes, such as those regulating cholesterol intake, synthesis, breakdown, and excretion, makes renal cell carcinoma very different from other cancers. Similar to cholesterol, fatty acid metabolism in ccRCC is also abnormal.

Acyl-CoA cholesterol acyltransferase1 (ACAT1/SOAT1) catalyzes the transfer of acyl groups from acyl-CoA to cholesterol to produce cholesterol ester, which is the main form of cholesterol when stored in cells and transported in plasma (95) (**Figure 2**). A study has shown that *ACAT1* mRNA and protein levels are decreased in ccRCC, and this transcriptional inactivation is significantly (84) associated with advanced pathological staging and short survival time of ccRCC (96). Another study that analyzed *ACAT1* by weighted co-expression network analysis in ccRCC concluded that *ACAT1* expression was decreased in high-grade ccRCC, and aggressive tumors were unable to obtain sufficient energy from ketolysis and fatty acid oxidation to support their growth (97). Some studies have also shown that silencing *ACAT1* or using *ACAT1* inhibitors such as ATR-101, avasimibe can effectively inhibit tumor cell growth (98–100).

The obesity paradox has been found in many forms of cancer, including diabetic kidney disease (9, 101, 102). Obesity increases the risk of clear cell renal cell carcinoma, but obese ccRCC patients seem to live longer than non-obese patients, which is known as the obesity paradox (103). It is speculated to be related to the accumulation and metabolism of lipids in ccRCC cells (104). An epidemiological and genomic study of the obesity paradox in ccRCC found that the higher the body mass index (BMI), the lower the cancer-specific mortality. Moreover, a genome-wide survey using BMI found that the expression of metabolic genes and fatty acid genes are different, which further supports the obesity paradox (105). This study extended the principle of the obesity paradox to the genomic level and provided us with many new ideas on fat accumulation in ccRCC: Is lipid accumulation in ccRCC cells related to the good prognosis of obese patients? Obesity is a prognostic indicator of ccRCC. Can the obesity paradox be used to improve patient outcomes? We hypothesized that the development and prognosis of renal cancer is closely related to the obesity paradox because one of the characteristics of ccRCC cells is the accumulation of lipids, leading to the occurrence of a large number of lipid droplets in the cells.

## Ferroptosis in ccRCC

As mentioned above, there are a large number of lipid droplets in the ccRCC cells. If these lipid droplets only exist in the cells, they will not be connected with the development of ccRCC. Therefore, we speculate that these lipid droplets play a role in disease progression.

Ferroptosis is a newly discovered form of iron-dependent oxidative cell death. We believe that this is closely related to lipid metabolism. Unlike traditional apoptosis and necrosis, it is characterized by the fatal accumulation of lipid reactive oxygen species (ROS), which suggests that genes expressing abnormal lipid metabolism play a very important role in the process of ferroptosis (106). This must be correlated with the accumulation of lipid droplets in ccRCC cells.

There are three main pathways of ferroptosis:

1. Regulation of the glutathione (GSH)/GPX4 (glutathione peroxidase 4) pathway, including sulfur metabolism and glutamine pathways.
2. The pathway of iron metabolism which includes the autophagy related-protein - Atg7- nuclear receptor coactivator 1 pathway, p62 - Kelch-like ECH-associated Protein 1- nuclear factor erythroid-2 related factor 2 pathway related to ferritin metabolism.
3. Pathways related to lipid metabolism, such as p53-SAT1 (spermidine/spermine N1-Acetyltransferase 1) -ALOX15 (Arachidonate 15-Lipoxygenase), acyl-CoA synthetase long-chain family member 4 (ACSL4), and lysophosphatidylcholine acyltransferase 3 (LPCAT3), are responsible for lipid regulation and ferroptosis (107).

Because the ccRCC metabolic mechanism is highly dependent on glutamine, one of the most important pathways in ferroptosis, the GSH/GPX4 pathway directly affects the proliferation of ccRCC cells. ccRCC cells use the metabolite of glutamine and glutathione to combat ROS generated by lipid peroxidation (the mechanism of glutamine and glutathione in ccRCC will be mentioned later). In other words, glutathione is an important means of combating ferroptosis (12). Lipid metabolism pathways are also related to cancer progression. *ACSL4* uses long-chain polyunsaturated omega-6 fatty acids to build cell membranes. This process plays a key role in the synthesis of cell membranes and is an important pathway for ferroptosis. Studies have shown that the regulation of *ACSL4* gene expression in breast cancer cells is related to the sensitivity of cancer cells to ferroptosis (108). ALOX15 oxidizes polyunsaturated fatty acids to produce biologically active lipid metabolites, such as lipid peroxides. Therefore, it is a downstream enzyme of the p53-SAT1-ALOX15 pathway and one of the key enzymes responsible for ferroptosis. It has also been speculated that the expression level of *ALOX15* is negatively correlated with renal cancer grade (109). LPCAT3 maintains the systemic lipid homeostasis by regulating intestinal lipid-absorbing lipoprotein secretion and lipogenesis in the liver (110). Studies have found that *LPCAT3* gene expression is upregulated in ccRCC. This is mainly because ccRCC cells need to synthesize a large amount of phospholipids to build cell membranes and proliferate in large quantities (111). Therefore, we speculated that there is a strong correlation between ferroptosis and lipid metabolic pathways in ccRCC. The correlation between ferroptosis and lipid metabolic pathways requires further research. Erastin, considered an efficient inducer of ferroptosis, can mediate ferroptosis through multiple molecules, such as the cystine-glutamate transporter receptor and p53 (112). RCC cells are more susceptible to erastin-induced cell death than other cells. Further studies demonstrated that erastin induced RCC cell death in a manner characteristic of iron drape disease (increased lipid ROS production and decreased GPX4 expression) and was inhibited by antioxidants (10).

Current studies have found that ferroptosis is closely related to the death of RCC cells. A study on the correlation between Hippo-Yes Associated protein/TAZ (PDZ-binding motif) pathway and ferroptosis in renal cancer cells showed that TAZ knockout led to resistance to ferroptosis in RCC cells, while the overexpression of TAZS89A led to sensitivity of cells to ferroptosis (113).

We still cannot fully summarize the influence of ferroptosis on ccRCC, but we can be certain that it is one of the pathways that may be taken as an effect of lipid metabolism and glutamine metabolism in ccRCC.

## The Role of Lipid Droplets in ccRCC

As the most significant pathological feature of ccRCC, the existence of lipid droplet accumulation not only reveals the abnormal lipid metabolism of ccRCC, but also indicates some death pathways of ccRCC cells. It has been pointed out that lipid droplets in ccRCC cells can be used as bioenergy fuel and raw materials for cell membrane generation, and can also be involved in endoplasmic reticulum stress (114). Therefore, we are very interested in the formation and role of lipid droplet itself.

Fatty acids are substrates for the synthesis of various lipid types. Diacylglycerol acyltransferase (DGAT) catalyzes diacylglycerol to synthesize triacylglycerol (TAG) with FAs and plays an important role in lipid synthesis (115) (**Figure 2**). *DGAT1* and *DGAT2*, two genes encoding DGAT, have been identified, and their main functions have been recognized in the past ten years. Researchers have used molecular tools to study the metabolic changes in mice lacking these two enzymes, revealing the function of *DGAT1/2 (* 116). Currently, *DGAT1/2* is known to play a positive regulatory role in many cancers related to lipid accumulation (117–120). Inhibitors targeting *DGAT* activity have also made significant progress (121–123). Related studies have tentatively confirmed the important role of *DGAT* in cancer, and we hope that more relevant studies can describe the detailed mechanism of *DGAT* in ccRCC, including other types of RCC.

Fatty acid binding protein (FABP) is responsible for transferring fatty acids to specific cell chambers and plays an important role in lipid binding and transport. Many types of FABP comprise the FABP family (124) (**Figure 2**). Studies have shown that the locations where FABPs perform overlapping functions are not limited to the synthesis and transport of lipids. They also exhibit unique characteristics in specific cells and tissues and their biochemical processes (125). By comparing the expression of *FABP* family genes in ccRCC samples and normal tissues, we found that the expression of *FABP5*, *6*, and *7* in ccRCC samples was significantly upregulated, while the expression of *FABP1* was significantly downregulated. This result shows that the influence of the FABP family on ccRCC is also diverse. Moreover, the prediction of overall survival or disease-free survival of ccRCC patients with high expression of *FABP5/6/7* and low expression of *FABP*1 was significantly reduced (126).

When we focus back on upstream genes, we find that lipid droplet formation is consistently influenced by the VHL-HIF pathway. We discussed earlier that the loss of *VHL* expression leads to the intake of glucose, which has been reported to be the raw material for lipid synthesis and is mediated by HIF. Carnitine palmitoyltransferase 1 (CPT1A) can transport fatty acids to mitochondria and reduce intracellular accumulation, while increased *HIF-1/2α* caused by reduced *VHL* expression inhibits this step (127). The report also found that CPT1A may serve as a therapeutic target for ccRCC.

In the ferroptosis section we mentioned that the presence of lipid droplets in ccRCC cells acts through oxidative stress, but focused on the ferroptosis process, which ultimately leads to cell death. However, oxidative stress has two sides. ROS can increase genomic instability and promote the occurrence of cancer (128). Therefore, the content of lipid droplets in ccRCC should maintain a balance, which can not only make the gene mutation lead to cancer, but also cannot lead to the generation of ferroptosis due to the excess of ROS. As a result, lipid droplets are not only produced, but also removed. It has been reported that microtubule-associated protein family 1-s(MAP1S) is used to activate the autophagy system of cells to remove the lipid droplets, and the removal of lipid droplets reduces ROS content to a level that is insufficient to affect gene stability and expression (129). The report suggests that the rational use of this mechanism is a new approach to the treatment of ccRCC.

In summary, due to the double-sided nature of oxidative stress, the presence of lipid droplets in ccRCC also has different effects. The general idea of current research is to break the balance of oxidative stress, so that it is more likely to cause ccRCC cell death or reduce ccRCC cell generation.

## Glutamine in RCC Cells

Glutamine as the raw material of lipid droplets (114) has always played an important role in RCC, and although it has been reported that the formation of lipid droplets in ccRCC is not required by glutamine (127), inhibitors of glutamine metabolism do inhibit the development of ccRCC. Therefore, it is necessary to discuss the effects of glutamine on ccRCC.

In the TCA cycle, glutamine is converted to glutamate by glutaminase (GLS), whereas the conversion of glutamate to α-ketoglutarate (α-KG), an intermediate product of the tricarboxylic acid cycle, can be catalyzed by transaminase or glutamate dehydrogenase (GDH) (11, 130). In cancer cells, glutamine is mainly transported to the mitochondria by SLC1A5 (solute carrier family 1 member 5) to participate in TCA (**Figure 1**). SLC1A5 overexpression induced by HIF-2α-mediated hypoxia in cancer cells plays a key role in cancer metabolic reprogramming (131). A recent study identified a small-molecule drug candidate, IMD-0354, that targets the glutamine transporter SLC1A5 to inhibit the uptake of glutamine by cancer cells, blocking an important energy source for cancer cells and thus, slowing their growth (132). They also found that small-molecule drugs can be used in combination with LDHA inhibitors and GLS1 inhibitors to inhibit cancer development.

Experiments showed that cancer cells with the Warburg effect were dependent on glutamine as a carbon source for TCA (133). This also explains why citrate, an intermediate product of TCA, can be used in lipid synthesis pathways as well as other biosynthetic pathways (134). The synthesis of acetyl-CoA, the precursor of lipid synthesis in cancer cells, relies on glutamine to sustain it. Concurrently, related studies have shown that glutamine is related to the activation of mTORC1, which senses glutamine and leucine and is activated by glutamine decomposition and α-ketoglutaric acid production upstream of Rag (11). Bis-2-[5-phenylacetamido-1,2,4-thiadiazol-2-yl] ethyl solubility (BPTES), which inhibits glutaminase activity, reduces glutamate and α-KG levels, and increases the intermediates of glycolysis (135). As a GLS inhibitor, it inhibits oncogenic transformation in IDH1-mutated glioma cells (136, 137). mTORC1 signaling is often activated by glutamine decomposition. Upon inhibition of glutamine decomposition by GLS inhibitors (such as BPTES 21) or siRNA targeting GLS or GDH, mTORC1 activation can be blocked theoretically to inhibit the progression of cancer (11, 130, 138). Metabonomic studies of ccRCC have shown a high uptake of glutamine in renal cancer (139, 140). At present, glutamine metabolism in the treatment of RCC is believed to be related to sunitinib resistance, and the glutamine transporter SLC1A5 in this study was significantly overexpressed in sunitinib-resistant samples compared to the control group (141).

Glutathione (GSH), the final metabolite of glutamine, also plays an important role in ferroptosis. Glutathione expression is significantly reduced in ferroptotic cells (10). GSH and its metabolise-related metabolites have been shown to be increased in patients with advanced ccRCC (142). In ccRCC, GSH reacts with excess reactive ROS in the body to produce oxidized glutathione, which achieves the function of antioxidant protection against oxygen free radical attack of cancer cells. A large amount of lipid accumulates in ccRCC, leading to a large amount of ROS depending on excessive lipid production, which is the premise of ferroptosis (143). Therefore, we can reduce the generation of GSH, a feature of ccRCC, to promote ferroptosis. At present, there are reports of system Xc^-^ inhibitors and GPx4 inhibitors that induce ferroptosis to achieve therapeutic effect (144, 145). In terms of ccRCC treatment, GPx1 was used as a biomarker to judge the prognosis of ccRCC (146), but there have been no reports on the application of ferroptosis in clinical treatment over a wide range. We believe that the research in this area has great potential and is a key direction for future drug development for ccRCC.

## Conclusion

RCC is considered to be more prone to metabolic disease. Its pathogenesis is similar to that of many other cancers. For example, cell proliferation requires large quantities of cholesterol and fatty acids, as well as the formation of large amounts of lactic acid and aerobic glycolysis with the incomplete utilization of glucose. Nevertheless, RCC exhibits unique characteristics. Compared with other cancers, the lipid metabolism process remains specific, mainly due to the diversity of lipid synthesis, uptake, and transformation in RCC cells. In recent years, several research results have had an impact on the publicly recognized cancer mechanism, and even the widely recognized Warburg effect has strong evidence that it is cognitively wrong. Current research on lipid metabolism in RCC mainly involves the transport receptors of fatty acids and cholesterol and many of their precursors, the related synthetase and rate-limiting enzymes that control synthesis, and the upstream genes that regulate the expression of these enzymes and receptor proteins. Several studies are more inclined to conduct follow-up experiments by analogy and rely on the research results and foundation pertaining to other types of cancers, such as breast and prostate cancer. We believe that starting from lipid metabolism, assessing the differences between RCC and other cancers, including cholesterol intake and synthesis, iron death, obesity paradox, etc., we will be able to find more differences between RCC and other cancers and perform targeted research. These abnormal phenomena can help us better understand RCC and study the essence of RCC more objectively.

## Author Contributions

GW conceived the review. QL and XC undertook the initial research. XQ was involved in writing and plotting. QW reviewed the manuscript. All authors contributed to the article and approved the submitted version.

## Funding

The PhD Start-up Fund of Liaoning Province from GW (2021-BS-209, Liaoning Province, 30000 CNY).

## Conflict of Interest

The authors declare that the research was conducted in the absence of any commercial or financial relationships that could be construed as a potential conflict of interest.

## Publisher’s Note

All claims expressed in this article are solely those of the authors and do not necessarily represent those of their affiliated organizations, or those of the publisher, the editors and the reviewers. Any product that may be evaluated in this article, or claim that may be made by its manufacturer, is not guaranteed or endorsed by the publisher.

## Glossary

ccRCC: clear cell renal cell carcinoma
VHL: von Hippel-Lindau
PI3K: phosphatidylinositol-3-kinase
Akt: protein kinase B
mTOR: mammalian target of rapamycin
TCA: tricarboxylic acid cycle
P53: protein 53
VEGF: vascular endothelial growth factor
TGF-β2: transforming growth factor-β2
ATP: adenosine triphosphate
MYC: myelocytomatosis oncogene
AMPK: adenosine 5’-monophosphate-activated protein kinase
HIF: hypoxia-inducible factor
LDLR: low-density lipoprotein receptor
GLUT: glucose transporter
LDHA: lactate dehydrogenase A
PDGF: platelet-derived growth factor
NADPH: nicotinamide adenine dinucleotide phosphate
NOX: NADPH oxidase
EGFR: epidermal growth factor receptor (EGFR)
TSC: tuberous sclerosis complex subunit
SCD1: stearoyl-CoA desaturase 1
FASN: fatty acid synthase
ACC: acetyl-CoA carboxylase
FA: fatty acid
SERBP: serpine mRNA binding protein
ACLY: ATP citrate lyase
ACSS2: acetyl-CoA synthetase 2
HMGCS: hydroxymethylglutaryl-CoA synthase
HMGCR: hydroxymethylglutaryl-CoA reductase
LXR: liver X receptor
ABCA1: adenosine triphosphate binding cassette transporter A-1
ACAT1: acyl-CoA cholesterol acyltransferase-1
BMI: body mass index
ROS: reactive oxygen species
GSH: glutathione
GPX4: glutathione peroxidase 4
SAT1: spermidine/spermine N1-Acetyltransferase 1
ALOX15: arachidonate 15-Lipoxygenase
ACSL4: acyl-CoA synthetase long-chain family member 4
LPCAT3: lysophosphatidylcholine acyltransferase 3
DGAT: diacylglycerol acyltransferase
TAG: triacylglycerol
FABP: fatty acid binding protein
CPT1A: carnitine palmitoyltransferase 1
MAP1S: microtubule-associated protein family 1-s
GLS: glutaminase
α-KG: α-ketoglutarate
GDH: glutamate dehydrogenase
SLC1A5: solute carrier family 1 member 5
GSH: glutathione
